# Abrupt but smaller than expected changes in surface air quality attributable to COVID-19 lockdowns

**DOI:** 10.1126/sciadv.abd6696

**Published:** 2021-01-13

**Authors:** Zongbo Shi, Congbo Song, Bowen Liu, Gongda Lu, Jingsha Xu, Tuan Van Vu, Robert J. R. Elliott, Weijun Li, William J. Bloss, Roy M. Harrison

**Affiliations:** 1 School of Geography Earth and Environment Sciences, University of Birmingham, Birmingham B15 2TT, UK.; 2 Department of Economics, University of Birmingham, Birmingham B15 2TT, UK.; 3 School of Public Health, Imperial College London, London W2 1PG, UK.; 4 Department of Atmospheric Sciences, School of Earth Sciences, Zhejiang University, Hangzhou 310027, China.

## Abstract

The COVID-19 lockdowns led to major reductions in air pollutant emissions. Here, we quantitatively evaluate changes in ambient NO_2_, O_3_, and PM_2.5_ concentrations arising from these emission changes in 11 cities globally by applying a deweathering machine learning technique. Sudden decreases in deweathered NO_2_ concentrations and increases in O_3_ were observed in almost all cities. However, the decline in NO_2_ concentrations attributable to the lockdowns was not as large as expected, at reductions of 10 to 50%. Accordingly, O_3_ increased by 2 to 30% (except for London), the total gaseous oxidant (O*_x_* = NO_2_ + O_3_) showed limited change, and PM_2.5_ concentrations decreased in most cities studied but increased in London and Paris. Our results demonstrate the need for a sophisticated analysis to quantify air quality impacts of interventions and indicate that true air quality improvements were notably more limited than some earlier reports or observational data suggested.

## Teaser

True air quality improvements during the COVID-19 lockdowns in global cities are more limited than we thought.

## INTRODUCTION

Air pollution (both indoor and outdoor) is the single largest environmental risk to human health globally, contributing to 8.8 million deaths in 2015 (*1*). The World Bank estimated that air pollution costs the global economy $3 trillion in 2015 (*2*). It has been suggested that poor air quality is correlated with a higher mortality rate from COVID-19 infection (*3*). Although a causal relationship between the two is difficult to confirm, air pollution contributes to respiratory and cardiovascular diseases and thus has the potential to cause increased COVID-19 death rates (*4*).

In response to the COVID-19 crisis, governments around the world introduced severe restrictions on behavior or lockdowns, which led to the cessation of a large swathe of economic activity and thus reduced air pollutant emissions (*5*). The rapid and unprecedented reduction in the economic activity provides a unique opportunity to study the impact of a global-scale natural intervention on air pollution, which offers insights for the prioritization of future clean air actions.

Many recent studies have explored impacts of the COVID-19 lockdowns on air quality. The most common approach is to undertake a simple statistical analysis that compares air quality before and after the lockdowns began or during the lockdowns with the same periods in previous years (*6*, *7*). Some studies also compared the air quality before and after lockdown started for periods with similar meteorological conditions (*8*). Satellite observations of NO_2_ have also been used to estimate the reduction in column NO_2_ due to the lockdowns (*3*, *9*–*11*).

A major caveat in a number of these studies is that meteorology moderates the link between emissions and pollutant concentrations, and so, weather changes can mask the changes in emissions on air quality (*12*–*14*). Such methods cannot explain the observed severe pollution events during the lockdowns in some cities (*15*–*17*). Comparisons of pollutant levels in 2020 with previous years may assume that air pollutant emissions have not changed over the past few years, which is often not the case, particularly in those cities where clean air policy actions are in place (*14*, *18*). Furthermore, air pollutant emissions change substantially from winter to spring; thus, a direct comparison of air pollutant concentrations before and during the lockdowns could also give unreliable results. Venter *et al.* (*9*) developed statistical models (regression) to estimate the impact of lockdowns on air quality in several countries. However, the performance of the regression was often limited with correlation coefficients as low as 0.2. He *et al.* (*19*) applied a “difference-in-difference” approach, which may provide a more accurate estimate of air quality improvement; this method assumes that the control cities are not subject to any impacts.

Air quality modeling can also decouple the effect of emission changes from meteorology (*20*, *21*) and is often applied for scenario analysis. A major challenge in evaluating the impacts of short-term interventions on real-world air quality is to estimate emission changes (*16*, *20*, *21*).

Machine learning offers an alternative and reliable method in quantifying changes in air quality due to emissions and meteorological factors (*12*–*14*). Myllyvirta and Thieriot (*22*) used a random forest (RF) method (*13*), which was developed for assessing long-term air quality changes, to estimate the short-term changes in NO_2_ and PM_10_ in Europe due to the COVID-19 lockdowns (see Materials and Methods).

The purpose of this study was to evaluate the impacts and implications of the natural experiment of the COVID-19 lockdowns in spring 2020 on air quality. To do this, we optimized a weather normalization technique based on Grange and Carslaw (*13*) and Vu et al. (*14*) to decouple the effects of meteorology from short-term emission changes on surface air quality monitoring data in 11 global cities that were subjected to extensive lockdown measures. The deweathered data allow us to quantitatively evaluate the real-world changes in air quality due to the lockdown measures in these cities (see Materials and Methods). These selected cities cover a range of air pollution climates, from highly to less polluted and from PM_2.5_- to NO_2_-dominated pollution. Data were divided into roadside, urban background, and rural sites to better understand the impacts of road traffic and urban emissions on air quality changes.

## RESULTS

**Fig. 1:**
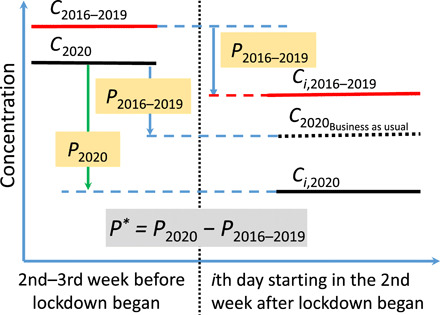
Concept of detrending air pollutant levels. *C*_2016–2019_ and *C*_2020_ are the average concentrations of an air pollutant in the second and third weeks before the lockdown start date or equivalent in 2016–2019 and 2020, respectively; *C*_*i*,2016–2019_ and *C*_*i*,2020_ are the daily average concentrations of an air pollutant in the *i*th day starting in the second week after the lockdown start date or equivalent in 2016–2019 and 2020, respectively. The vertical dashed line represents lockdown start date. *P*_2016–2019_ and *P*_2020_ are the percentage changes in air pollutant levels after versus before the lockdown began or equivalent in 2016–2019 and 2020, respectively (see Eq. 2 in the main text for definition). *C*_2020_Business as usual__ is the hypothetical concentration for the *i*th day starting in the second week after the lockdown date under “business-as-usual” (i.e., no lockdown) conditions. This is calculated from the prelockdown concentration (*C*_2020_) assuming the same percentage change as in 2016–2019 (*P*_2016–2019_, as the “business-as-usual” change). The detrended percentage change P* (i.e., the change in air pollutant concentration arising from lockdown effects alone) is given by *P*_2020_ − *P*_2016–2019_.

We first estimated the percentage change (*P*) in the observed or deweathered concentrations of air pollutants using the following equation

$$P=\bar{(C_{i}-C)/C\times 100\%}$$ (1)

where *C* is the average concentration in the second and third weeks before the lockdown date or equivalent (as a prelockdown baseline), and *C_i_* is the average concentrations in the *i*th day (from the 1st to 28th day) starting in the second week after the lockdown start date for each city and for each year (see Fig. 1). For example

$$P_{2020}=\bar{(C_{i,2020}-C_{2020})/C_{2020}\times 100\%}$$ (2)

The week immediately before and after the lockdown date was considered a transition period and so was excluded in the calculations. We recognize that the transition may have started earlier in some cities such as London, but for consistency, we applied the same Eq. 1 for calculation. For clarification, we will use *P*_obs_ and *P*_dew_ to represent changes in observed and deweathered concentrations, respectively.

We then estimated the detrended percentage change (P*) in the concentration of each air pollutant (deweathered only; Fig. 1), calculated by

$$P^{*}=P_{2020}–P_{2016-2019}$$ (3)

where *P*_2020_ and *P*_2016–2019_ are percentage changes in deweathered concentrations of air pollutants in 2020 and 2016–2019, respectively. P* was calculated by Monte Carlo simulations (*n* = 10,000) based on the normal distribution of *P*_2020_ and *P*_2016–2019_.

P* removes the “business-as-usual” variability in concentrations from winter to spring (i.e., 2016–2019 as a baseline, *P*_2016–2019_) and thus represents the change attributable to lockdown measures. This business-as-usual variability can be caused by changes in anthropogenic activities (e.g., domestic heating) and natural processes [e.g., biogenic volatile organic compound (VOC) emissions]. For example, when the domestic heating demand reduces in local spring, there may be less local emissions of air pollutants; as a result, the concentrations of air pollutants are lower if emissions from other major sources do not increase and meteorological conditions are similar.

### Changes in NO_2_, O_3_, and O*_x_*

**Fig. 2:**
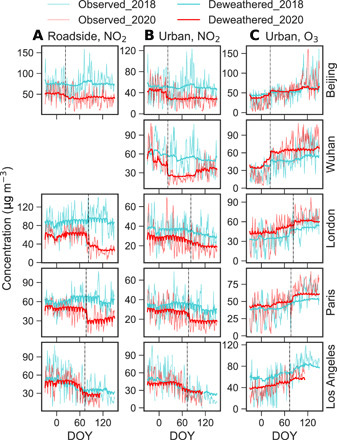
Observed and deweathered daily NO_2_ and O_3_ concentrations in selected cities before and after the lockdown start dates or equivalent in 2020 versus 2018. Columns correspond to (**A**) NO_2_ at roadside sites, (**B**) NO_2_ at urban background sites, and (**C**) O_3_ at urban background sites; rows show different cities as indicated. Fine and heavy lines indicate observed and deweathered concentrations, respectively. Data are shown from December to May, shown as day of year (DOY; 1 January = 1), where the vertical dashed lines represent lockdown date. The sudden drop in deweathered NO_2_ and corresponding increase in deweathered O_3_ are apparent in Beijing, Wuhan, and Paris, whereas London and Los Angeles show more gradual changes. The saw-like shape in the deweathered data in some cities captures the weekly cycles of NO_2_ and, to a lesser extent, O_3_, particularly in western cities. Results from other cities/sites are shown in fig. S1. No data are available for roadside sites in Wuhan.

Observed NO_2_ levels are highly variable, with daily concentrations changing notably during the study period (Fig. 2 and fig. S1). Pollution events (e.g., spikes in Fig. 2) appeared repeatedly during the lockdowns, such as in Beijing, Wuhan, and Paris. Observed NO_2_ at roadside sites decreased substantially in all cities after the lockdowns began, with *P*_obs_ ranging from −29.3 ± 33.1% in Berlin to −53.5 ± 18.9% in London (table S1); observed NO_2_ at urban background sites also decreased substantially, with the *P*_obs_ ranging from −10.1 ± 36.6% in London to −60.2 ± 14.8% in Delhi; and observed NO_2_ at rural sites increased in London (*P*_obs_ = +115.8 ± 90.2%) and Paris (*P*_obs_ = +99.2 ± 66.7%) but decreased in other cities after lockdown started.

Deweathered NO_2_ usually shows a similar pattern to the observations, but the magnitudes and sometimes even the signs of changes are different. A sudden drop, distinct from the data in 2018, is clearly observed at urban sites in 2020 after the lockdowns began in all cities except London and Los Angeles, which show a more gradual change (Fig. 2 and fig. S1). This confirms that the sudden changes in 2020 are indeed due to the lockdown measures.

**Fig. 3:**
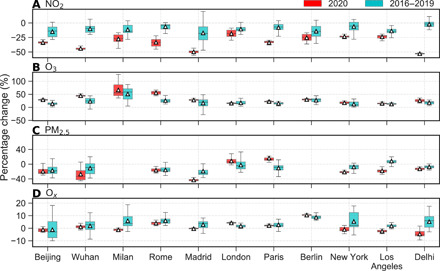
Box plots of percentage change (*P*_dew_) in deweathered concentrations of air pollutants in 2020 versus 2016–2019. Rows represent (**A**) NO_2_, (**B**) O_3_, (**C**) PM_2.5_, and (**D**) O*_x_*. Lower and upper box boundaries represent the 25th and 75th percentiles, respectively; line and triangle inside boxes represent median and mean values, respectively; lower and upper error lines represent 1.5 * IQR (interquartile range) below the third quartile and above the first quartile, respectively. Number of samples for *P*_dew_ in 2020 and 2016–2019 is usually 28 and 112, respectively.

Deweathered NO_2_ at urban background sites in 2020 decreased in all cities after the lockdowns began, with *P*_dew_ ranging from −18.2 ± 6.0% in London to −52.9 ± 1.4% in Delhi (Table 1); deweathered NO_2_ at roadside sites decreased more markedly in most cities (Fig. 2, fig. S1, and table S2). We also noticed that deweathered NO_2_ (*P*_dew_) in 2016–2019 decreased in almost all cities from winter to spring, although the magnitude of change is usually much smaller than in 2020 (Fig. 3). Thus, the absolute values of the detrended NO_2_ change, P*, is smaller than the corresponding *P*_dew_. Table 1 shows that the decline in NO_2_ due to the lockdown measures at urban background sites is mostly less than 30% in the studied cites.

**Table 1:** Percentage changes (%) in deweathered (*P*_dew_) and detrended (P*) NO_2_, O_3_, PM_2.5_ mass concentrations, and O*_x_* mixing ratios at urban background sites in the studied cities. See Eqs. 1 to 3 for definition of *P*_dew_ and P*. N.A., data not available.

		**Beijing**	**Wuhan**	**Milan**	**Rome**	**Madrid**	**London**	**Paris**	**Berlin**	**New York**	**Los Angeles**	**Delhi**
NO_2_	*P*_dew_	−33.4 ± 2.2	−43.9 ± 2.2	−27.4 ± 8.3	−33.2 ± 6.1	−49.7 ± 3.1	−18.2 ± 6.0	−33.6 ± 3.3	−25.4 ± 6.0	−23.3 ± 2.0	−23.8 ± 3.4	−52.9 ± 1.4
	P*	−18.5 ± 9.2	−33.9 ± 7.3	−16.3 ± 11.4	−27.1 ± 7.7	−35.2 ± 21.3	−7.7 ± 7.7	−25.8 ± 7.1	−11.3 ± 13.1	−17.0 ± 8.3	−9.9 ± 6.1	−51.0 ± 5.2
O_3_	*P*_dew_	28.9 ± 2.0	44.5 ± 3.4	66.8 ± 29.2	55.8 ± 6.7	28.0 ± 3.8	15.8 ± 1.8	22.2 ± 2.4	29.9 ± 3.0	17.4 ± 3.9	14.8 ± 2.2	26.2 ± 5.8
	P*	14.8 ± 5.3	21.8 ± 13.6	15.4 ± 37.6	29.8 ± 10.1	11.2 ± 18.3	−1.6 ± 8.1	7.0 ± 5.1	2.6 ± 8.9	5.3 ± 9.8	2.3 ± 5.3	8.2 ± 8.4
^PM^_2.5_	*P*_dew_	−19.3 ± 9.6	−27.0 ± 18.7	N.A.	−16.4 ± 5.2	−43.1 ± 3.4	8.6 ± 8.3	16.5 ± 10.7	N.A.	−21.5 ± 2.6	−18.0 ± 5.4	−12.7 ± 2.8
	P*	−2.4 ± 14.7	−15.7 ± 24.8	N.A.	−0.7 ± 9.6	−24.1 ± 18.4	10.9 ± 16.7	27.4 ± 15.3	N.A.	−13.9 ± 6.9	−40.3 ± 26.9	−5.2 ± 4.8
O*_x_*	*P*_dew_	−1.1 ± 2.0	1.1 ± 1.4	−1.3 ± 1.0	4.1 ± 1.2	−0.4 ± 1.3	4.2 ± 0.8	2.1 ± 0.6	10.5 ± 0.8	−1.0 ± 1.8	−2.3 ± 1.0	−4.6 ± 3.5
	P*	0.1 ± 7.4	−0.7 ± 4.4	−7.2 ± 5.9	−2.0 ± 2.9	−3.4 ± 3.2	−2.4 ± 1.3	−0.6 ± 2.3	1.8 ± 1.9	−6.4 ± 6.8	−4.1 ± 1.7	−9.8 ± 6.3

Deweathered NO and NO*_x_* (=NO + NO_2_) in 2020 dropped more markedly (table S2) after the lockdown began than was observed for NO_2_. For example, *P*_dew_ values for NO and NO*_x_* at urban background sites in London were approximately −24.8 ± 6.3 and −21.0 ± 5.9%, respectively, whereas that for NO_2_ was −18.2 ± 6.0%. At roadside sites in London and Rome, deweathered NO*_x_* decreased by more than 50% during the lockdowns, a much larger change than that for NO_2_ (−47.0% in London and −35.2% in Rome).

In contrast to changes in NO_2_, observed O_3_ at roadside sites in 2020 increased in all cases (fig. S1 and table S1) after the lockdown began, with the *P*_obs_ values ranging from +19.5 ± 21.0% in Madrid to +155.6 ± 83.2% in Milan. Observed O_3_ at urban/rural sites also increased during the lockdowns (fig. S1). A sudden increase in deweathered O_3_ after the lockdown began was observed in most of the cities (Fig. 1 and fig. S1). The *P*_dew_ values (Table 1 and table S2) for deweathered O_3_ range from +15.0 ± 3.0% in Los Angeles to +128.5 ± 41.9% in Milan at roadside sites, from +14.8 ± 2.2% in Los Angeles to +66.8 ± 29.2% in Milan at urban background sites, and from +1.5 ± 0.9% in London to +57.9 ± 6.3% in Milan at rural sites. However, there is an increasing trend in O_3_ levels at urban background sites during the same periods in 2016–2019 (Figs. 2 and 3), with *P*_dew_ values ranging from +12.1 ± 9.1% in New York to +51.2 ± 23.4% in Milan (auxiliary data table S1). As a result, the detrended O_3_ changes (P*) at urban background sites are much smaller than those of the corresponding *P*_dew_ values; there is an obvious increase in P* in Beijing, Wuhan, Milan, and Rome, but a small change or even a decrease in other cities (Table 1).

**Fig. 4:**
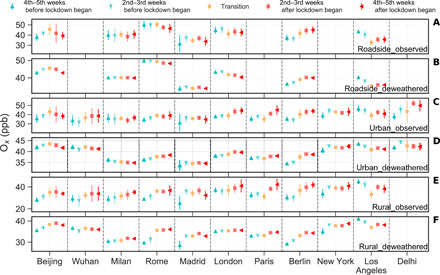
Observed and deweathered O*_x_* (i.e., NO_2_+ O_3_) mixing ratios in the 5 weeks before and after the lockdown start dates in the studied cities in 2020. The six rows (from top to bottom) show results from roadside observed (**A**) and deweathered (**B**), urban background observed (**C**) and deweathered (**D**), and rural observed (**E**) and deweathered (**F**) mixing ratios. Deweathered O*_x_* shows little change before and after the lockdown dates in 2020 and is similar across all urban background sites (all close to 40 ppb). Error bars (included for all points) represent 1 SD (*n* = 14). Transition period refers to the 2 weeks with the lockdown start date in the middle.

Accordingly, the observed levels of total gaseous oxidant (i.e., O*_x_* = NO_2_ + O_3_), a parameter unaffected by the titration reaction between NO and O_3_ but representing net photochemical production of O_3_, showed a different pattern to NO_2_ and O_3_, with little change before and during the lockdowns, whether at roadside, urban background, or rural sites (Fig. 4). Observed O*_x_* at urban background sites in 2020 range from 35.0 ± 5.4 parts per billion (ppb) in Madrid to 44.8 ± 8.5 ppb in Delhi during the 10-week period with lockdown start date in the middle. Deweathered O*_x_* mixing ratios at urban background sites were remarkably similar across the cities, at approximately 40 ppb (Fig. 4). Only a small change in deweathered O*_x_*, before and after lockdown started in 2020, was observed at urban background sites in all the cities, with *P*_dew_ values ranging from −4.6 ± 3.5% in Delhi to +10.5 ± 0.8% in Berlin (Table 1). Small changes were also seen during the same periods in 2016–2019, with *P*_dew_ for deweathered O_3_ ranging from −1.2 ± 7.1% in Beijing to +8.7 ± 1.7% in Berlin (Fig. 2 and auxiliary data table S1). Detrended O*_x_* at urban background sites generally decreased during the study period in most of the cities, but the absolute change is relatively small, i.e., mostly within ±5%; changes at rural sites are more variable with almost half of the cities showing a slight increase (table S3).

### Changes in PM_2.5_ and PM_10_

**Fig. 5:**
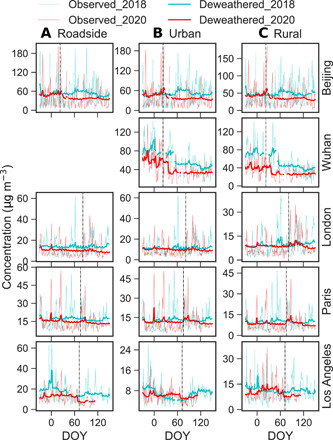
Observed and deweathered daily PM_2.5_ concentrations in the selected cities before and after the lockdown start dates or equivalent in 2020 versus 2018. Columns correspond to (**A**) roadside, (**B**) urban background, and (**C**) rural sites; rows show different cities as indicated. Fine and heavy lines indicate observed and deweathered concentrations, respectively. Data are shown from December to May, shown as day of year (1 January = 1), where the vertical dashed lines represent lockdown start date. Results from other cities/sites are shown in fig. S2. No data are available for roadside sites in Wuhan.

Figure 5 and fig. S2 show that the average observed PM_2.5_ levels in 2020 reduced after lockdown started in the two more polluted cities, Wuhan and Delhi. No clear changes were observed in other cities, particularly when comparing levels to those in previous years (Fig. 5 and fig. S2). In Beijing, Paris, and London, pollution events were observed after the lockdowns began (Fig. 5). Unlike NO_2_, the peak levels observed during the lockdowns were sometimes even higher than those before lockdown began (e.g., London). The *P*_obs_ values for observed PM_2.5_ in 2020 range from −40.8 ± 28.4% in Los Angeles to +107.6 ± 148.5% in London at roadside sites, from −38.6 ± 17.2% in Madrid to +152.9 ± 165.0% in London at urban background sites, and from −34.2 ± 26.8% in Delhi to +164.5 ± 148.7% in London at rural sites (table S1).

Deweathered PM_2.5_ in 2020 showed a clearer pattern than that apparent in the observations (Fig. 5). Unlike deweathered NO_2_ and O_3_, a sudden decrease in PM_2.5_ after lockdowns started was not detected in most of the cities, with the exceptions of Wuhan and Rome (fig. S1). However, sudden decreases were observed in some cities (such as Los Angeles, New York, Beijing, and Wuhan) a few days after or before the lockdowns began. Figure 5 and fig. S2 show that the deweathered PM_2.5_ before the lockdown began in 2020 was similar to that in 2018 in Beijing, lower in Wuhan, London, Paris, and Berlin, but higher in Rome and Delhi. In Beijing, there was an increase in deweathered PM_2.5_ after the lockdown began initially, but there was a decrease afterward (Fig. 5). Deweathered PM_2.5_ in London and Paris also increased after the lockdowns began, but in contrast, there was no obvious decrease even 3 weeks from the lockdown date.

The changes in deweathered PM_2.5_ are similar at different site types (Fig. 5 and fig. S2). Deweathered PM_2.5_ at roadside sites in 2020 increased slightly during the lockdowns by +1.0 ± 7.2% in London and +0.2 ± 9.1% (*P*_dew_) in Paris but decreased with changes (*P*_dew_) ranging from −2.8 ± 1.3% in New York to −37.8 ± 4.8% in Los Angeles (table S2). A similar trend is also observed in the deweathered PM_2.5_ at urban background and rural sites (Fig. 5 and fig. S2). An obvious decrease in deweathered PM_2.5_ at urban background sites during the same study periods was also observed in 2016–2019 in some cities but not in others (Fig. 3 and auxiliary data table S1). The detrended change (P*; Table 1) in PM_2.5_ at urban background sites shows a decrease in Los Angeles (−40.3 ± 26.9%), Madrid (−24.1 ± 18.4%), Wuhan (−15.7 ± 24.8%), New York (−13.9 ± 6.9%), and Delhi (−5.2 ± 4.8%), but little changes or even increases in the other cities.

The overall patterns of variations in observed and deweathered PM_10_ (fig. S4) are similar to those of the PM_2.5_ (fig. S3). A slight difference in some cities is that there were more variabilities/contrasting patterns at different types of site. For example, a larger decline in *P*_dew_ for deweathered PM_10_ at roadside sites than that at urban background sites is observed in Beijing, Madrid, London, Paris, and Berlin (table S2), potentially reflecting a coarse particle source from road traffic (e.g., non–exhaust emissions) (*23*). Furthermore, in Los Angeles and Delhi, the decline in deweathered PM_10_ is significantly larger than that of PM_2.5_ whether at urban background or rural sites (table S2), implying a reduced contribution of coarse particles to PM_10_.

### Changes in CO and SO_2_

Deweathered CO levels were lower after lockdown started than before in 2020. This pattern is different from that in 2018 (fig. S4). A sudden change is observed in Rome and Wuhan only. In Beijing, deweathered CO increased slightly after the lockdown began, before falling for about 2 weeks, after which there was a substantial increase at all three types of sites. Thereafter, the deweathered CO decreased substantially, ~40% (*P*_dew_) lower than that during the same period in 2018. In New York (roadside sites), a decline in deweathered CO is observed a week after the lockdown began. In Delhi, the decreasing trend in deweathered CO at urban background sites is not distinguishable from that in 2018, whereas at rural sites, CO clearly declined from a few days before the lockdown began.

The change in deweathered SO_2_ after the lockdowns began is dependent on the site or city (fig. S4). No sudden change is observed in any of the cities immediately after the lockdowns. In Beijing, deweathered roadside and urban SO_2_ increased initially and then decreased by ~20%. In all cases, the deweathered SO_2_ concentration in 2020 is much lower than that in 2018. In London, deweathered SO_2_ declined for a few days before the lockdown began at roadside sites. Deweathered SO_2_ in Wuhan and Rome decreased about a month before the lockdowns but did not change during the lockdowns. In New York (roadside sites), a decline in deweathered SO_2_ is observed a week after the lockdown began. Delhi saw a substantial decrease in deweathered SO_2_ about 2 weeks after the lockdown started, and the decrease in deweathered CO at urban background sites is not distinguishable from that in 2018, whereas at rural sites, it clearly declined from a few days before the lockdown began.

## DISCUSSION

The deweathered and detrended data are used to understand how the air quality responded to the changes in activity associated with the COVID-19 lockdowns of early 2020 and the potential implications of such interventions for developing future air pollution abatement strategies and thus improving human health.

### The importance of deweathering and detrending

Large differences between the deweathered and observed concentrations of air pollutants were observed in the studied cities (Fig. 2 and figs. S1 to S3). Observed daily average NO_2_ concentrations are much higher than the deweathered ones during some periods. Our estimated NO_2_ decline in Wuhan due to lockdown effects is much lower than that estimated by Le *et al.* (*15*), who reported up to 93% reduction in NO_2_ in Wuhan during the lockdown. If we look at the observations only, we can indeed see >90% decrease from the peak concentration before the lockdown began to the lowest one afterward (Fig. 2), but this is mainly due to changes in meteorological conditions, not emissions. The observed PM_2.5_ also exhibited remarkable meteorologically driven variability regardless of cities or site types and sometimes differed by more than a factor of 3 when compared with deweathered concentrations (Fig. 5 and fig. S2). In general, major differences are apparent between the observed and deweathered results when the meteorological conditions change substantially over the study period. For example, the changes in observed and deweathered PM_2.5_ at urban background sites in Beijing before and after the lockdown began were +19.2 ± 108.6% (*P*_obs_) and −19.3 ± 9.6% (*P*_dew_), respectively. In this case, emission reductions and the unfavorable meteorological conditions drove changes of approximately −19.3 and +38.6% in the observed levels, respectively, leading to an overall +19.2% increase in PM_2.5_. Our results demonstrate that meteorological variations, rather than emission changes on the scale of those occurring during the COVID-19 lockdowns, dominate short-term variability in air pollutant concentrations, which is consistent with previous studies (*12*, *14*, *20*, *24*).

Apart from deweathering, detrending the “business-as-usual” changes is also crucial in estimating real changes attributable to interventions (i.e., lockdowns). In the “business-as-usual” scenario, air pollutant emissions (both anthropogenic and natural) and, thereby, concentrations may change from winter to spring, whether there is a lockdown or not (see 2016–2019 data in Fig. 3). For example, a general increase in deweathered O_3_ is observed from winter to spring in 2016–2019 in all the studied cities (Figs. 2 and 3 and fig. S1). Such an increase reflects changing photochemical steady-state partitioning from NO_2_ to O_3_ (Northern Hemisphere cities moving into spring with increased solar radiation intensity and day length), alongside wider increases in photochemical ozone formation, enhanced by increased emission and chemical reactivity of VOCs. Taking this “business-as-usual” variability into account, the detrended percentage changes (P*) in O_3_ are much smaller than the corresponding *P*_dew_ (Table 1 and table S3). Not accounting for this seasonality would lead to a different conclusion, as by Sicard *et al.* (*7*), that O_3_ concentration increased substantially in response to the lockdowns.

In some cities, there are considerable variabilities in *P*_dew_ in 2016–2019 (Fig. 3). This may be partly due to specific events such as holidays around the lockdown dates, leading to a decrease in air pollutant emissions for a particular year. In this instance, the absolute value of *P*_dew_ in 2016–2019 could be slightly overestimated, and thus that of the P* underestimated. However, because we included 4 years of data (2016–2019) for detrending, the impact of a specific event on the P* values is small.

Our detrended results (Table 1 and table S3) demonstrate that the decreases in NO_2_ and increases in O_3_ due to the COVID-19 lockdowns are not as large as previous studies have reported (*7*, *21*) or as the raw observational data show (table S1). Note also that anthropogenic air pollutant emissions reduce year by year, such as in London and Beijing, as a result of clean air policy actions and vehicle fleet evolution (*14*, *18*). Thus, the approach widely used in the literature to estimate the lockdown effects by subtracting NO_2_ during the equivalent periods in earlier years from that in 2020 (*6*, *7*, *11*, *15*) may also overestimate the effects attributed to the lockdowns (Fig. 3 and Table 1).

Considering urban background sites in Wuhan (a widely studied city) as an example, observed NO_2_ and O_3_ changed by −47.3 ± 17.4% and +166.5 ± 60.5% (*P*_obs_ values, obtained from unadjusted concentration data before/during lockdown; table S1), values similar to those reported by Shi and Brasseur [−54 ± 7% and + 220 ± 20% (*25*)]; changes of approximately −51.8 and +40.0% are obtained by subtracting NO_2_ and O_3_ concentrations during the second to fifth weeks after the lockdown dates in 2016–2019 from those in 2020 (i.e., without adjustment for meteorology), values which are similar to those reported by Sicard *et al.* [−57 and +36% (*7*)]. Our estimated changes in deweathered NO_2_ and O_3_ (*P*_dew_) are −43.9 ± 2.2% and +44.5 ± 3.4%, which are similar to those reported by Zhao *et al.* [−51.7 and +58% (*21*)]. However, these estimations (*7*, *21*, *25*) are considerably higher (sometimes by a factor of 10) than our detrended results (P*), which are −33.9 ± 7.3% for NO_2_ and +21.8 ± 13.6% for O_3_. This may at least partially explain why the estimated changes in NO_2_ and O_3_ due to the lockdown effects in the studied cities reported here are lower than those published elsewhere (*7*, *11*, *15*, *21*, *25*), and demonstrate the necessity of disentangling the changes due to meteorological variation and seasonality and from the lockdown-driven changes in emissions to understand the resulting differences in air pollutant concentrations.

### Drivers of changes

The deweathered NO_2_ showed a sudden decrease after the lockdown began in most of the cities (Fig. 2 and fig. S1). Detrended NO_2_ at urban background sites declined the most in Delhi (−51.0 ± 5.2%), Madrid (−35.2 ± 21.3%), and Wuhan (−33.9 ± 7.3%) (Table 1). A given reduction in NO*_x_* emission, and hence NO*_x_* abundance, is expected to lead to a smaller reduction in ambient NO_2_ levels, as the fast NO*_x_*-O_3_ photochemistry shifts the NO_2_/NO*_x_* ratio in favor of NO_2_. The fact that the NO*_x_* changes are larger than those of NO_2_ supports this argument (tables S1 and S2). A substantially larger decline in NO*_x_* and NO_2_ was observed at roadside than at urban background sites, suggesting that the decline in NO_2_ during the lockdowns is largely driven by changes in road traffic as the dominant source of NO*_x_* in urban atmosphere (*16*). Mobility data from Google Maps (https://google.com/covid19/mobility/) suggest that traffic volumes reduced by 60 to 80% in the cities considered here. However, this mobility decrease does not correspond directly to the same reduction in road traffic–related NO*_x_* emissions. For example, in London, although private car use reduced by about 80%, heavy good vehicles (HGVs) on the road only reduced by 30 to 40%. It is possible that if the change in the number of HGVs, which account for a smaller percentage of total vehicle population but a large proportion of vehicular NO*_x_* emissions (*26*, *27*), is small, then the changes in total road traffic emissions of NO*_x_* may be much smaller than expected. Decreases in activity levels from other combustion sources, such as power plants and industry (*22*), may have contributed to the decline in NO_2_, at least in some cities, as shown by the small decline in SO_2_ in some cities (fig. S4). Such changes are difficult to quantify, but the much smaller (and absence of any sudden) changes in SO_2_ compared with NO_2_ (Fig. 2 and fig. S2)—as indicated by the increase in SO_2_/NO_2_ ratio in Wuhan, London, Paris, Rome, and Delhi (auxiliary data table S1)—suggest that reductions in NO*_x_* emissions from stationary sources were less than those from traffic emissions. This is consistent with Le Quéré *et al.* (*5*), who estimated that in Europe and the United States, electricity use reduced by 9 and 5%, respectively. Note also that domestic emissions may have increased with an increase in people working or studying from home. We recognize that our methodology is unable to attribute the actual changes in emissions on a sector-by-sector basis. This could be revisited in the future when emission inventories for the spring 2020 lockdown period are developed and evaluated against observations.

O_3_ is a secondary pollutant, and its variation is driven by several factors. Dominant among these is the NO*_x_*-O_3_ photochemical steady state. The decrease in NO (tables S1 and S2) led to reduced O_3_ titration, through which reductions in traffic-related NO emissions translate directly into increases in O_3_, relative to the prelockdown period; the time constant for this NO*_x_*-O_3_ interaction in daylight is of the order of minutes. The fact that deweathered O_3_ increased suddenly after the lockdown began and that changes in deweathered NO were more pronounced than those in NO*_x_* and NO_2_, particularly at roadside sites (fig. S1 and table S2), support this well-understood atmospheric chemistry (*28*). This effect—of a reduced urban decrement in O_3_—will be partially offset by reductions in primary NO_2_ emissions from traffic and, on a much longer time scale (hours to days, rather than minutes), by net O_3_ production. Under an extreme condition, if all traffic-related NO*_x_* emissions are assumed to be NO, O*_x_* would remain unchanged in response to lockdown-driven changes in traffic (but NO_2_ would decrease, and O_3_ would increase). In reality, primary NO_2_ emissions from road traffic decreased during the lockdowns, so O*_x_* should fall. Detrended O*_x_* fell slightly at roadside and urban background sites in most of the cities (Table 1 and table S3). Detrended O*_x_* increased at rural sites in some of the cities (table S3), which indicates an increase in net photochemical production of O_3_ at some of the studied sites (*28*). The different pattern of changes in detrended O_3_ represents a nonlinear response of O_3_ formation rates to the (relative) changes in NO*_x_* and VOC emissions, depending on the prevailing O_3_ production regime at each location, but usually with a greater impact downwind of conurbation locations (*29*, *30*).

Drivers of the response of PM_2.5_ levels to the lockdown measures are more complex since both primary emissions and secondary formation contribute to PM_2.5_ in ambient air. Deweathered PM_2.5_ reduced after the lockdowns began at urban background sites in most of the cities, including Wuhan, Rome, New York, Los Angeles, and Delhi (fig. S2). This could be explained by (i) the expected reductions in primary emissions of PM_2.5_ and its gaseous precursors (e.g., NO_2_, SO_2_, and VOCs) during the lockdowns and (ii) limited change in the formation rate of secondary aerosol as shown by the small variation in PM_2.5_/CO ratio (fig. S5).

Deweathered PM_2.5_ increased in London and Paris for an extended period (more than 3 weeks) after the lockdowns began (Fig. 5). It also increased in Beijing after the lockdown began, although for a shorter period. One possible explanation for this unexpected result is that enhanced secondary aerosol formation overwhelmed the reduced primary PM_2.5_ emissions. In Chinese megacities, secondary particles typically contribute to >50% of PM_2.5_ mass (*31*, *32*). In London, secondary aerosols contribute roughly half of PM_2.5_ at roadside sites, increasing to ~90% of PM_2.5_ at rural sites, with the contribution lying between these values at urban background sites (*33*). Such contributions are even larger during pollution events (*15*, *16*, *31*). Thus, changes in PM_2.5_ are often driven by variations in secondary aerosols, particularly during pollution events. In Beijing, Sun *et al.* (*34*) noted that primary aerosol decreased by 30 to 50%, while secondary inorganic aerosol and secondary organic aerosol (SOA) increased by 60 to 110% and 52 to 175%, respectively, during the early periods of the lockdown in 2020. The fact that substantial increases in PM_2.5_/PM_10_ (Paris) or PM_2.5_/CO (London; fig. S5) ratios accompanied the increase in deweathered PM_2.5_ (Fig. 5 and fig. S2) also supports the greater role of secondary aerosol during the study period in Paris and London. Zhao *et al.* (*35*) suggested that SOA formation depends nonlinearly on the ratio of VOCs to NO*_x_*; reduction in NO*_x_* emissions may lead to increased production of SOA given imbalanced emission abatement of NO*_x_* and VOCs. Le *et al.* (*15*) indicated that multiphase chemistry and enhanced atmospheric oxidative capacity drove haze events in China during the lockdowns. Huang *et al.* (*16*) also suggested that increase in oxidative capacity during lockdown in China/Beijing caused the observed air pollution events; however, the changes in deweathered O*_x_* levels (*P*_dew_) at urban background sites are rather small: Beijing (−1.1 ± 2.0%), London (+4.2 ± 0.8%), and Paris (+2.1 ± 0.6%) (table S2).

Another possible explanation is associated with changes in long-range transport, which brings air pollutants from nonlocal sources and thus contributes to the increase in deweathered PM_2.5_. In theory, the RF models should have normalized the impacts from long-range transport by including back-trajectory clusters. However, the model may not be able to perfectly reproduce secondary formation processes arising from long-range transport if there were limited cases to learn from, especially as such events tend to be episodic in nature. In this case, the model will treat pollution events arising from long-range transport as if there are higher emissions; this attribution will be retained during deweathering. This will cause uncertainties in the model. More observational data and modeling are needed to fully understand the phenomenon of increases in PM_2.5_ in London, Paris, and Beijing during the lockdowns. However, it is clear that a small reduction in primary PM_2.5_ emissions (e.g., from vehicular emission changes during lockdown) could be readily overwhelmed by enhanced secondary formation and/or PM_2.5_ transported from more polluted regions.

In Wuhan, the deweathered PM_2.5_ decreased to a small degree during the 2 weeks after the lockdown began (Fig. 5). However, the deweathered PM_2.5_/CO increased during the lockdowns (fig. S5), which suggests that enhanced secondary pollution (*36*) offsets the benefits of the reduction in primary emissions during the first 2 weeks of the lockdown. Thereafter, the deweathered PM_2.5_ did decrease more significantly (*P*_dew_ = −27.0 ± 18.7%). Similarly, in Beijing, the deweathered PM_2.5_ decreased 2 weeks after the lockdown began, so overall *P*_dew_ is negative (−19.3 ± 9.6%). These results suggest that if the reduction in emissions of gaseous precursors is sufficiently large, it should eventually lead to an overall decline in PM_2.5_. Such a hypothesis should be tested with chemical transport models with up-to-date emission inventories when these are available.

### Implications for future air pollution control

Our results demonstrate that restrictions on economic activities, particularly traffic, brought an immediate decline in detrended NO_2_ in all the studied cities. If similar levels of restriction were to have remained in place, the annual average NO_2_ concentration would comply with the air quality guidelines from the World Health Organization (WHO) (i.e., 40 μg m^−3^ for annual NO_2_) for the cities considered under average meteorological conditions, except for a limited number of roadside sites. However, the detrended percentage decline (i.e., attributed to lockdown effects) in NO_2_ is mostly below 30%. This is lower than the expected decline, partly due to the NO*_x_*-O_3_ photochemical steady state (converting NO to NO_2_), alongside seasonal effects, and partly due to the still important emissions of NO*_x_* from stationary and mobile pollution sources. Detrended O_3_ increased in most cities. This adds to the complexity of air pollution control, considering the potentially adverse impacts of O_3_ on human (*37*, *38*) and environmental health, including crop yields (*39*).

PM_2.5_ exhibited a more complex response to the lockdown measures. PM_2.5_ did not show an immediate decline to the lockdown measures except in Wuhan, Rome, and Los Angeles, even at the roadside sites. This is not too unexpected considering the relatively small contribution of road traffic to primary PM_2.5_ in most of the cities studied here and a large contribution from secondary sources (*16*, *31*). In China, much of the recent decrease in PM_2.5_ came from the reductions in residential solid fuel use and industrial activity rather than traffic emissions (*18*, *40*). Nevertheless, a decrease in deweathered PM_2.5_ is observed in most of the cities.

In Delhi, Wuhan, and Beijing, annual average PM_2.5_ concentrations are so far in exceedance of the WHO guideline (10 μg m^−3^) that the decline is far from sufficient to bring levels into compliance. Even in those cities where the annual average PM_2.5_ is close to 10 μg m^−3^, such as London and Paris, emission reductions on the scale of the spring 2020 COVID-19 lockdown measures may still be insufficient to bring concentrations into compliance with the current WHO guidelines. In addition, the frequent PM_2.5_ pollution events during the lockdowns in some cities, such as Beijing, London, and Paris, showed that actions of a magnitude similar to the lockdown measures are far from sufficient to avoid episodic pollution events in these cities. The mechanisms driving such changes have been explored in more detail by recent studies (*15*–*17*, *20*).

Li *et al.* (*41*) suggested that aggressive reductions in NO*_x_* and aromatic VOC emissions should be particularly effective for decreasing both PM_2.5_ and O_3_ in China. The huge reduction in NO*_x_* (fig. S3) and VOCs (*16*) in response to the COVID-19 lockdowns did reduce PM_2.5_ pollution in Beijing and Wuhan, but detrended O_3_ increased substantially (Table 1), at least up until mid-May. A slower pace of VOC emission reduction, relative to that for NO*_x_*, could risk a further increase in O_3_ pollution.

In summary, emission changes associated with the early-2020 COVID-19 lockdown restrictions led to complex and substantial changes in air pollutant levels, but the changes are smaller than expected. The decrease in NO_2_ will likely have benefits on public health, but the increase in O_3_ would counteract at least some of this effect (*37*, *38*). The magnitude and even the sign of changes in PM_2.5_ during the lockdowns differ significantly among the studied cities. Chemical processes of the mixed atmospheric system add complexity to efforts to abate secondary pollution (e.g., O_3_ and PM_2.5_) through reduction of precursor emissions (e.g., NO*_x_* and VOCs) (*42*). Future control measures will require a systematic approach toward NO_2_, O_3_, and PM_2.5_ tailored for specific cities, taking into account both primary emissions and secondary processes, to maximize the overall benefits to air quality and human health.

## MATERIALS AND METHODS

### Selected cities and data

Eleven cities were selected to ensure coverage of contrasting pollution climate: Beijing and Wuhan in China, Milan and Rome in Italy, Madrid in Spain, London in United Kingdom, Paris in France, Berlin in Germany, New York and Los Angeles in the United States, and Delhi in India. Of those, eight are capital cities. Wuhan was added because it was the first city where COVID-19 was reported and lockdown was first imposed. Milan was included because it is in northern Italy, one of the most seriously hit areas after Wuhan. In the United States, New York was the most seriously affected city, whereas Los Angeles was reported to have observed a greater decline in air pollution levels (*43*). All the study cities have been significantly affected by COVID-19 and implemented stringent lockdown measures to contain the COVID-19 pandemic in early 2020. Such measures were first implemented in Wuhan from 23 January 2020 and then 2 days later in all provinces in China (including Beijing). Tightened restrictive measures were implemented from 23 January 2020 in northern Italy, 13 March 2020 in the United States, 14 March 2020 in Spain, 17 March 2020 in France, 22 March 2020 in Germany, 23 March 2020 in the United Kingdom, and 25 March 2020 in India.

Site-specific hourly concentration of six criteria pollutants (PM_2.5_, PM_10_, O_3_, NO_2_, CO, and SO_2_) and other auxiliary pollutants (NO and NO*_x_*) from December 2015 to May 2020 were obtained from websites of local or national environmental agency or accredited third parties (table S4). In most cases, data from multiple stations for each site type are available. The NO_2_ concentrations reported from local governments, typically performed by the widely used molybdenum conversion/chemiluminescence method, may slightly overestimate true NO_2_ levels due to conversion of other labile N species to NO in the convertor stage. This problem is usually small for polluted urban sites but is larger for rural sites where overestimates of 17 to 30% have been reported (*44*). This is due to the conversion of NO*_x_* from primary sources to secondary nitrogen compounds during its transport toward more rural locations. Hence, concentrations reported as NO_2_ contain a small proportion of other NO*_y_* species, and the “true” NO_2_ levels would be lower than those officially reported, particularly at rural locations. We note that such uncertainties are effectively “built in” to monitor NO_2_ with respect to regulatory standards. NO*_x_* and NO data were obtained in cities where those data were publicly available. Data were usually downloaded from official sources, which are validated by the authorities. For those cities where data were not available from recognized official sources (i.e., Los Angeles and New York) at the time of access, we obtained the air quality data from the “OpenAQ” platform (https://openaq.org/). Data from Los Angeles were downloaded from the U.S. Environmental Protection Agency (USEPA) later (after the data analyses were done here), which were then compared with those from OpenAQ. We found that the site-specific data in Los Angeles from OpenAQ are highly correlated (slope = ~1, intercept = ~0) with those from USEPA. Air quality monitoring stations were selected to cover roadside, urban background, and rural sites when possible, and the site types were based on official classifications and maps. The downloaded data were screened and cleaned when necessary, following established methods (*24*).

The hourly temperature, relative humidity, atmospheric pressure, wind speed, and wind direction data for selected sites were obtained from the nearest meteorological observation site from the NOAA (National Oceanic and Atmospheric Administration) Integrated Surface Database (ISD) using the “worldmet” R package (https://CRAN.R-project.org/package=worldmet). In addition, hourly data for boundary layer height, total cloud cover, surface net solar radiation, and total precipitation at the selected sites were downloaded from the ERA5 reanalysis dataset (ERA5 hourly data on single levels from 1979 to present). For each site, 72-hour back trajectories at an hourly resolution were calculated using the Hybrid Single-Particle Lagrangian Integrated Trajectory (HYSPLIT) model. The starting height was set as 100 m to ensure that the receptor was aloft but remained within the boundary layer throughout the study period. The back trajectories were then clustered into 12 clusters using the Euclidian distance by “openair” R package (https://CRAN.R-project.org/package=openair). Those clusters were used to represent the common air masses that the sites were exposed to.

Observations at the air quality stations are used for official compliance purpose. Although these stations were built to represent the specific environment of the city (i.e., roadside, urban background, and rural), there may be some variabilities in the concentrations of air pollutants at different stations of the same type. This could cause potential uncertainties in our analyses if to represent the whole city. In this study, wherever possible, we used data from multiple stations for each site type (table S4), which reduced this uncertainty. Where only one station is available for a site type, the data may be subject to more influence from local emission sources. Therefore, what we reported here should be treated in the context of the site availability (see table S4). Furthermore, we would like to emphasize that our analyses focus on the high-resolution temporal variations, and thus, the trend will be broadly representative.

### RF model and weather normalization

Weather conditions change rapidly, causing variations in the concentration of air pollutants even when emissions do not change. Here, we applied a machine learning–based RF algorithm to decouple the effects of meteorological conditions. To do this, we first build an RF model for each pollutant and for each year (December to May). The RF model–based weather normalization technique was introduced in Grange *et al.* (*12*). Briefly, the RF model was built independently for each period (December 2015 to May 2016, December 2016 to May 2017, December 2017 to May 2018, December 2018 to May 2019, and December 2019 to May 2020), each pollutant, and each site type within a city. Seventy percent of the original data were randomly selected to build the model, which was then evaluated with the remainder (30%) of the dataset. Model performance for each pollutant and each time period (i.e., 2016–2020) is illustrated in fig. S6. Similar to Grange *et al.* (*12*, *13*) and Vu *et al.* (*14*), the performance of the models is usually very good, much better than that of regression models (*9*). The weather normalization was conducted using the “rmweather” R package, available at https://cran.r-project.org/web/packages/rmweather/index.html.

In the Grange *et al.* (*12*) approach, a new dataset of input predictor features including time variables (day of the year, day of the week, and hour of the day, but not the Unix time) and meteorological parameters (wind speed, wind direction, temperature, and relative humidity) is first resampled from the original observation dataset. Vu *et al.* (*14*) modified the default method to investigate the seasonal variations in trends for comparison with trends in primary emissions, by only resampling the weather variables (not the time variables). Specifically, weather variables at a specific hour of a particular day in the input datasets were generated by randomly selecting from the historical weather data (past 30 years) at the particular hour of different dates within a 4-week period (i.e., 2 weeks before and 2 weeks after that selected date). The two methods are fit for their own purposes but were not used here because (i) Grange *et al.* (*12*) normalized the diurnal and seasonal variations of the primary emissions, which is unrealistic in the real world, and (ii) although Vu *et al.* (*14*) provided diurnal and seasonal variations of the primary emissions, this is inappropriate in detecting short-term emission interventions because the normalized concentrations for a particular hour of a Julian day were not comparable with those from the different hour of a different Julian day, considering that they were resampled from different weather datasets, which would be affected by different seasonal weather conditions.

To address those limitations and better investigate the impacts of short-term lockdown on air quality, we applied a mixed method. We only normalized the weather data but not time variables, similar to Vu *et al.* (*14*), and resampled from the whole study period, similar to Grange *et al.* (*12*). The improved method is more suitable for tracking emission changes. The input features for the model included time variables (i.e., Unix time, Julian day, day of the week, and hour of the day), meteorological data from surface observations (i.e., temperature, relative humidity, wind speed, wind direction, and atmospheric pressure), meteorological data from ERA5 reanalysis dataset (i.e., boundary layer height, total cloud cover, surface net solar radiation, and total precipitation), and air mass clusters based on the HYSPLIT back trajectories. The day of week and air mass clusters were categorical variables, while all others were numeric. Following Vu *et al.* (*14*), the parameters for the RF models are as follows: a forest of 300 trees, n_tree = 300; the number of variables that may split at each node, mtry = 3; and the minimum size of terminal nodes, min_node_size = 3. For every weather normalization, the explanatory variables were resampled (excluding the time variables) without replacement and randomly allocated to a dependent variable observation. The 1000 predictions were then aggregated using the arithmetic mean to obtain the deweathered concentration.

## Supplementary Material

http://advances.sciencemag.org/cgi/content/full/7/3/eabd6696/DC1

Adobe PDF - abd6696_SM.pdf

Abrupt but smaller than expected changes in surface air quality attributable to COVID-19 lockdowns

## SUPPLEMENTARY MATERIALS

Supplementary material for this article is available at http://advances.sciencemag.org/cgi/content/full/7/3/eabd6696/DC1
