# Supplementary material for: Abrupt but smaller than expected changes in surface air quality attributable to COVID-19 lockdowns
Source: Sci Adv. 2021 Jan 13;7(3):eabd6696. doi: 10.1126/sciadv.abd6696 (PMC7806219; doi:10.1126/sciadv.abd6696)
Supplement: http://advances.sciencemag.org/cgi/content/full/7/3/eabd6696/DC1 [file supp_7_3_eabd6696__index.html]

Science Advances | Science AdvancesAAASSearchScience AdvancesMenu

## Supplementary Materials

# Abrupt but smaller than expected changes in surface air quality attributable to COVID-19 lockdowns

Zongbo Shi, Congbo Song, Bowen Liu, Gongda Lu, Jingsha Xu, Tuan Van Vu, Robert J. R. Elliott, Weijun Li, William J. Bloss, Roy M. Harrison

Download Supplement

**This PDF file includes:**

- Figs. S1 to S6
- Tables S1 to S4
- Auxiliary data table S1

**Files in this Data Supplement:**

- Adobe PDF - abd6696\_SM.pdf
