# Supplementary material for: Abrupt but smaller than expected changes in surface air quality attributable to COVID-19 lockdowns
Source: Sci Adv. 2021 Jan 13;7(3):eabd6696. doi: 10.1126/sciadv.abd6696 (PMC7806219; doi:10.1126/sciadv.abd6696)
Supplement: http://advances.sciencemag.org/cgi/content/full/7/3/eabd6696/DC1 [file supp_7_3_eabd6696__1.pdf]

Science Advances
AAAS

advances.sciencemag.org/cgi/content/full/7/3/eabd6696/DC1

Supplementary Materials for

Abrupt but smaller than expected changes in surface air quality attributable to COVID-19 lockdowns

Zongbo Shi*, Congbo Song*, Bowen Liu, Gongda Lu, Jingsha Xu, Tuan Van Vu, Robert J. R. Elliott, Weijun Li, William J. Bloss, Roy M. Harrison

*Corresponding author. Email: z.shi@bham.ac.uk (Z.S.); c.song.1@bham.ac.uk (C.S.)

Published 13 January 2021, *Sci. Adv.* **7**, eabd6696 (2021)
DOI: 10.1126/sciadv.abd6696

This PDF file includes:

Figs. S1 to S6
Tables S1 to S4
Auxiliary data table S1

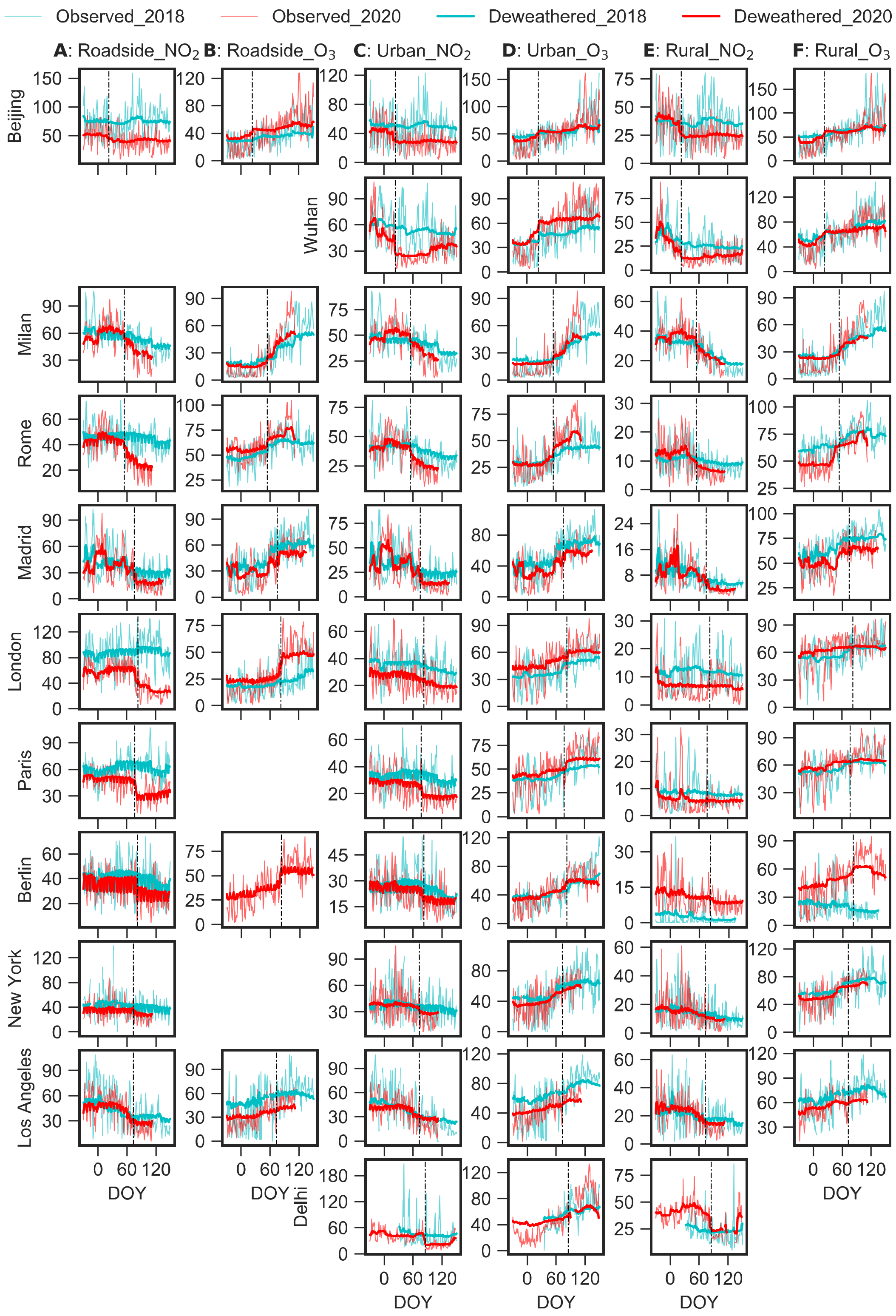

Fig. S1. Observed and deweathered daily concentrations (unit: μg m^{-3}) in the studied cities before and after the lockdown start dates or equivalent for NO_2 and O_3 in 2020 versus 2018. Columns correspond to roadside NO_2 (**A**) and O_3 (**B**); urban background NO_2 (**C**) and O_3 (**D**); and rural NO_2 (**E**) and O_3 (**F**); rows show different cities as indicated. Fine and heavy lines indicated observed and deweathered concentrations, respectively. Data are shown from December to May, shown as Day of Year (DOY; 1 January = 1), where the vertical dashed lines represent lockdown date. No NO_2 and O_3 data available for roadside sites in Wuhan and Delhi and no O_3 data for roadside sites in Paris and New York and rural sites in Delhi.

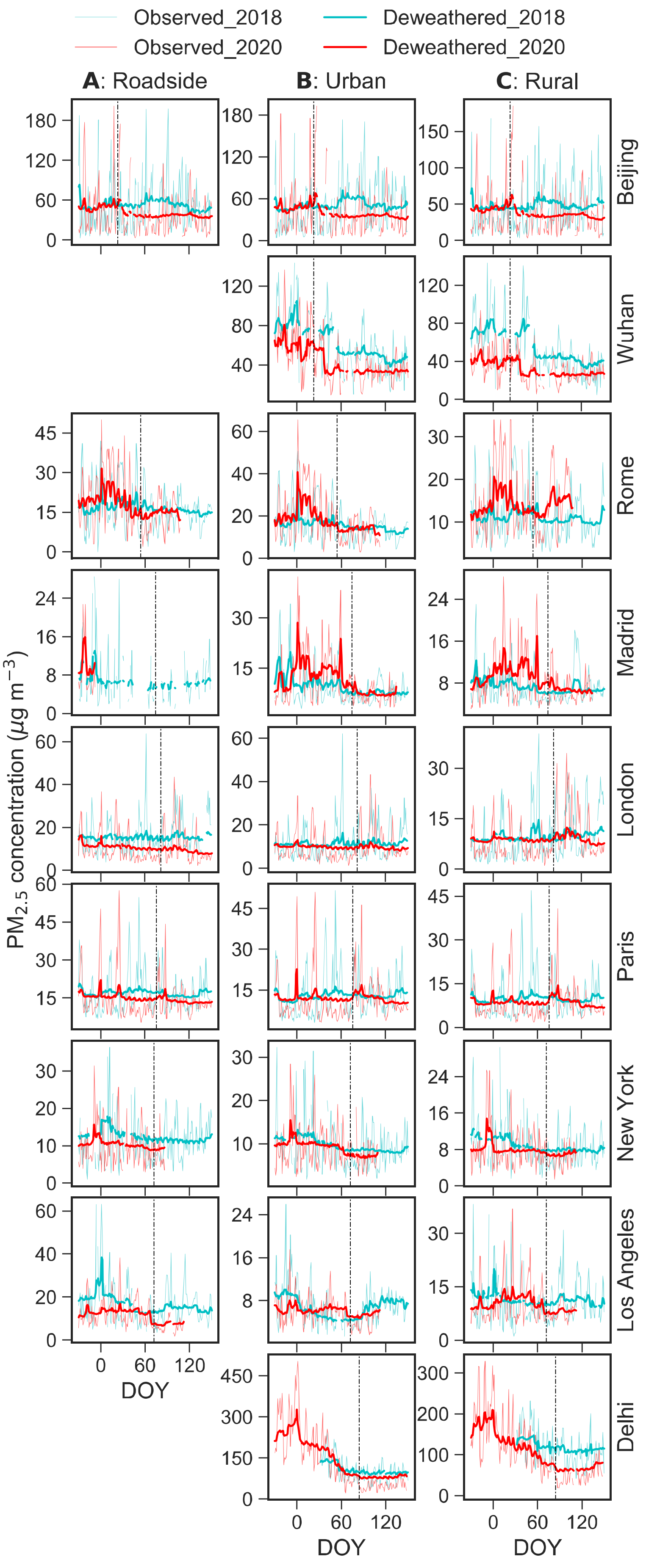

Fig. S2. Observed and deweathered daily $PM_{2.5}$ concentrations (unit: μg m^{-3}) in the studied cities before and after the lockdown start dates or equivalent in 2020 versus 2018. Columns correspond to roadside (**A**), urban background (**B**) and rural sites (**C**), respectively; rows show different cities as indicated. Fine and heavy lines indicated observed and deweathered concentrations, respectively. Data are shown from December to May, shown as Day of Year (DOY; 1 January = 1), where the vertical dashed lines represent lockdown date. No data available for roadside sites in Wuhan and Delhi.

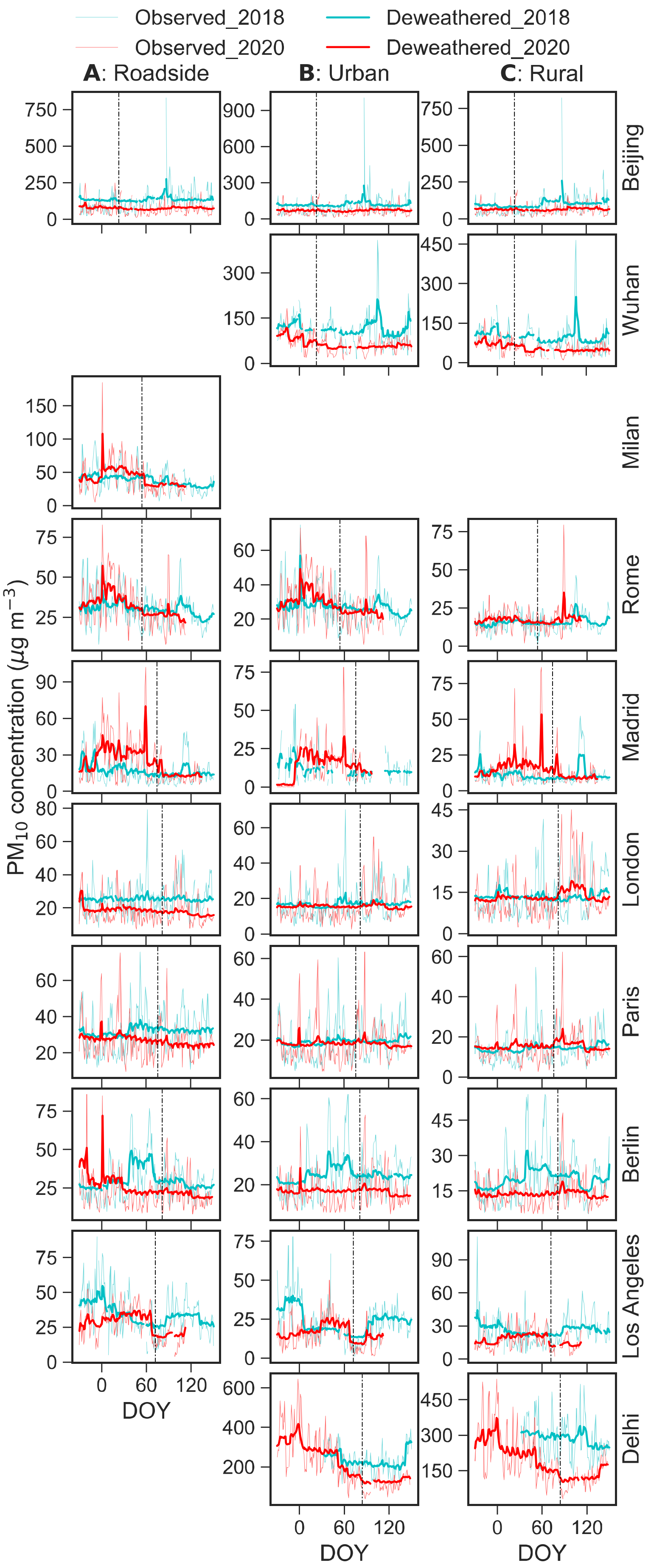

Fig. S3. Observed and deweathered daily PM_{10} concentrations (unit: µg m^{-3}) in the studied cities before and after the lockdown start dates or equivalent in 2020 versus 2018. Columns correspond to roadside (**A**), urban background (**B**) and rural sites (**C**), respectively; rows show different cities as indicated. Fine and heavy lines indicated observed and deweathered concentrations, respectively. Data are shown from December to May, shown as Day of Year (DOY; 1 January = 1), where the vertical dashed lines represent lockdown date. No data available for roadside sites in Wuhan and Delhi and for urban background and rural sites in Milan.

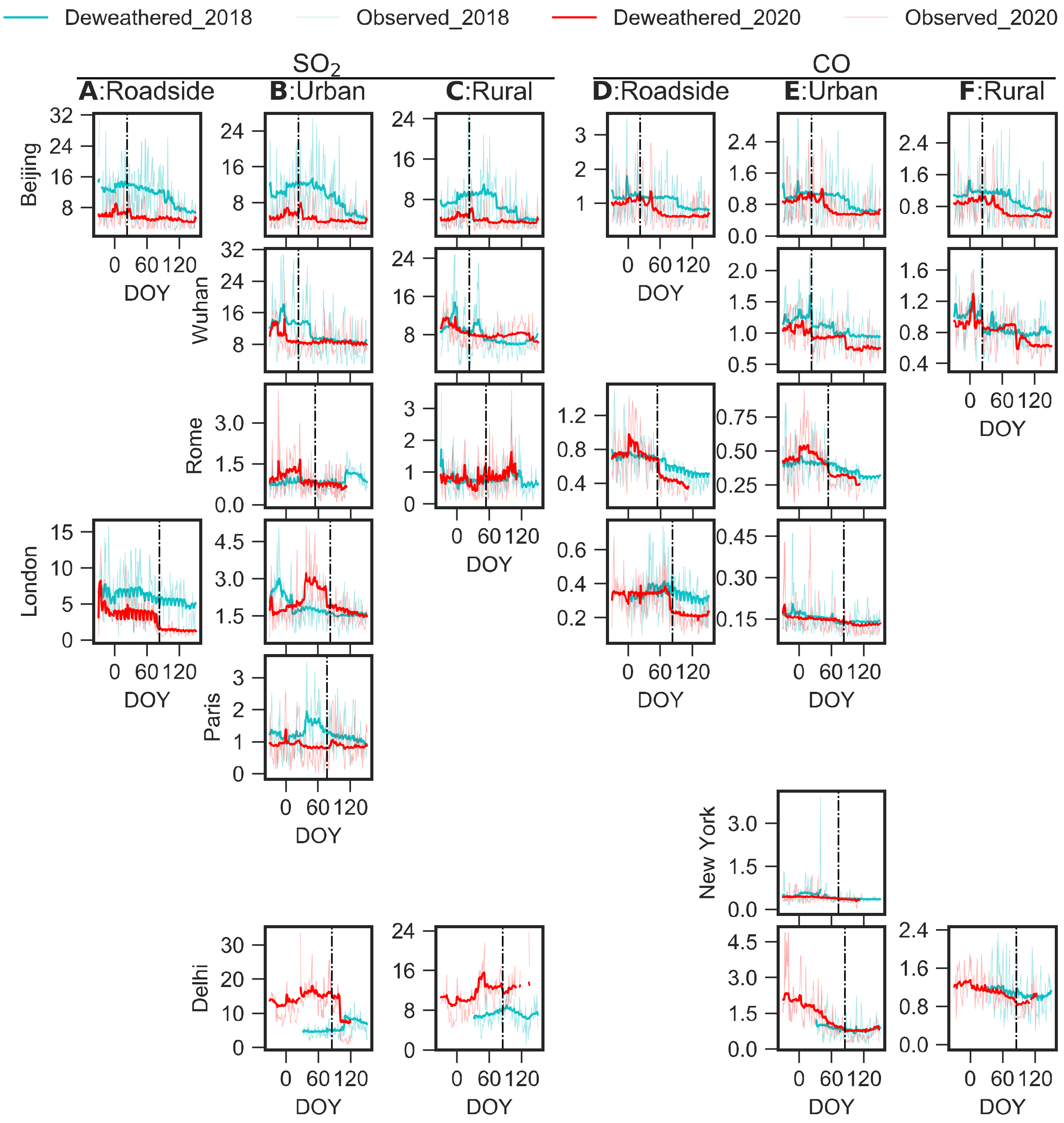

Fig. S4. Observed and deweathered daily concentrations before and after the lockdown start dates or equivalent for SO_2 (μg m^{-3}) and CO (mg m^{-3}) in 2020 versus 2018. Columns correspond to SO_2 concentrations at roadside (**A**), urban background (**B**) and rural sites (**C**), CO concentrations at roadside (**D**), urban background (**E**) and rural sites (**F**), respectively; and rows show different cities as indicated. Fine and heavy lines indicated observed and deweathered concentrations, respectively. Data are shown from December to May, shown as Day of Year (DOY; 1 January = 1), where the vertical dashed lines represent lockdown date. No data available for a number of sites / cities.

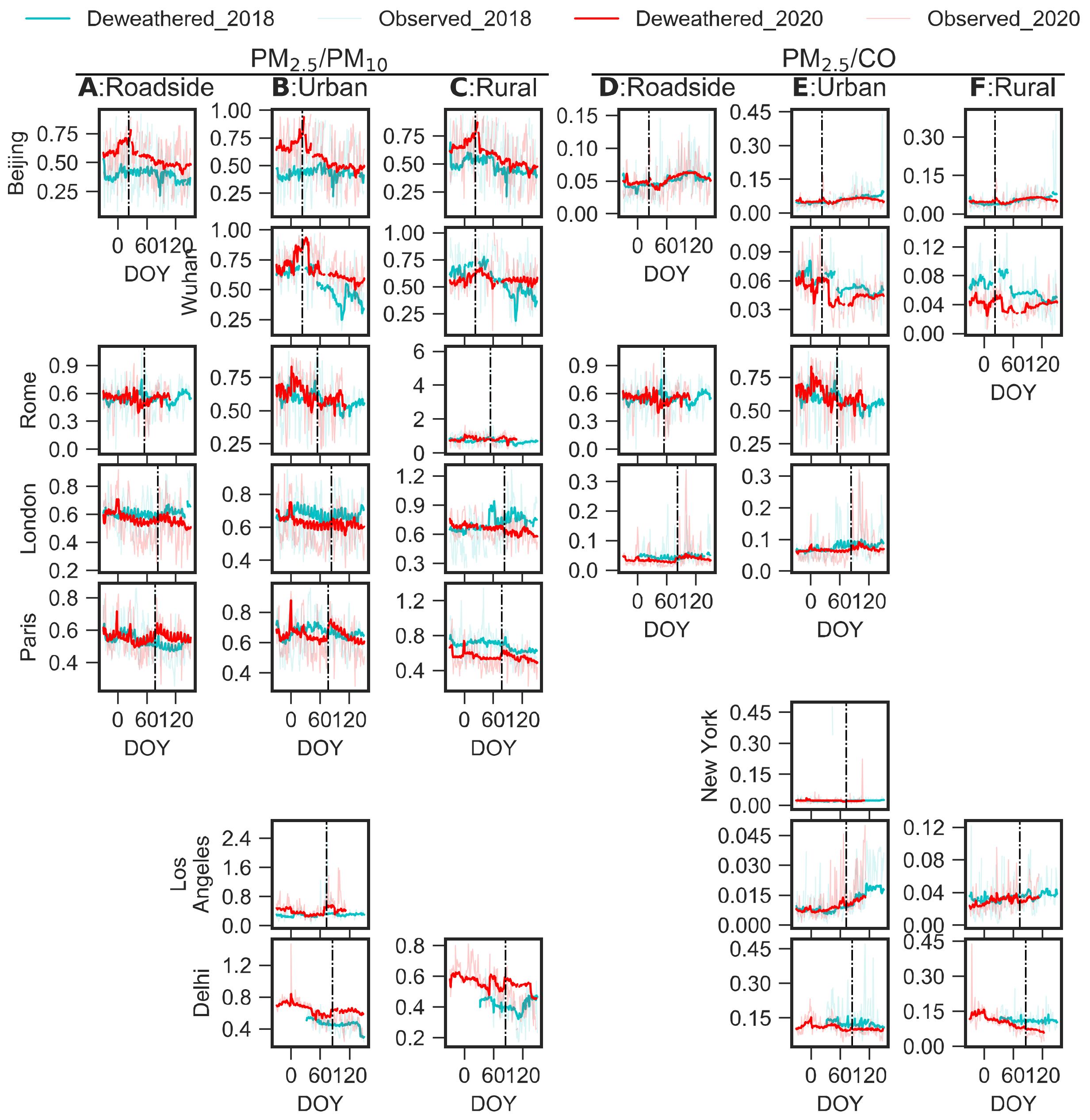

Fig. S5. Observed and deweathered $PM_{2.5}/PM_{10}$ and $PM_{2.5}/CO$ ratios before and after the lockdown start dates or equivalent in 2020 versus 2018. Columns correspond to $PM_{2.5}/PM_{10}$ ratio (µg m^{-3}/µg m^{-3}) at roadside (**A**), urban background (**B**), and rural sites (**C**); $PM_{2.5}/CO$ ratio (µg m^{-3}/µg m^{-3}) at roadside (**D**), urban background (**E**) and rural sites (**F**); rows show different cities as indicated. Fine and heavy lines indicated observed and deweathered concentrations, respectively. Data are shown from December to May, shown as Day of Year (DOY; 1 January = 1), where the vertical dashed lines represent lockdown date. No data available in a number of sites / cities.

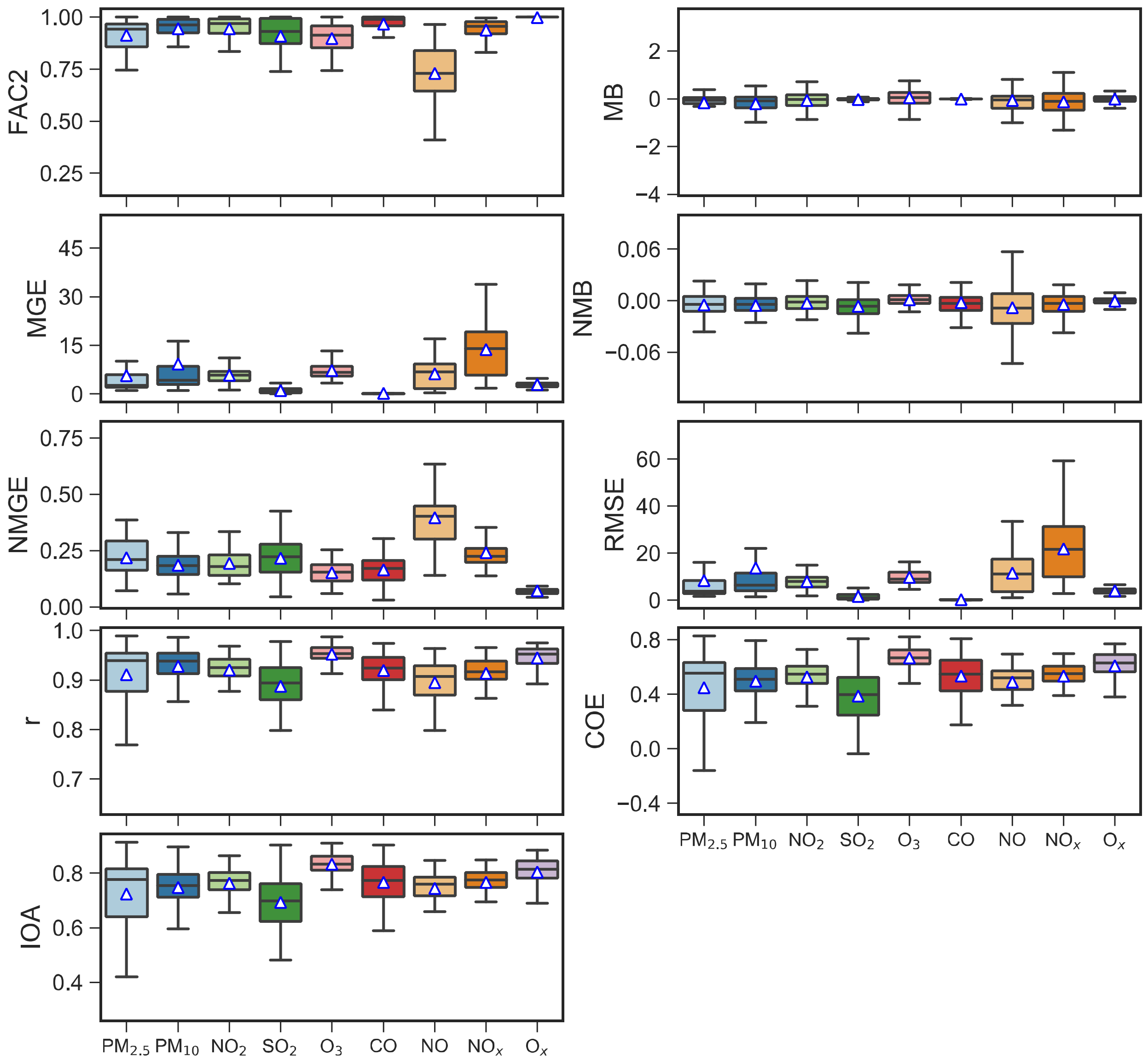

Fig. S6: Boxplots of the model performance parameters for testing data set of each air pollutant (in hourly time resolution) in 2016-2020. Note: FAC2 (fraction of predictions with a factor of two), MB (mean bias), MGE (mean gross error), NMB (normalized mean bias), RMSE (Root-mean-square deviation), NMGE (normalized mean gross error), r (correlation coefficient), COE (Coefficient of Efficiency), and IOA (Index of Agreement). Lower and upper box boundaries represent 25th and 75th percentiles, respectively; line and triangle inside box represent median and mean, respectively; lower and upper error lines represent 1.5*IQR (interquartile range) below the third quartile and above the first quartile, respectively. The average ($\pm$ standard deviation) correlation coefficients (r) are 0.91 ± 0.07 for $PM_{2.5}$, 0.91 ± 0.04 for PM_{10}, 0.92 ± 0.03 for NO_2, 0.89 ± 0.06 for SO_2, 0.95 ± 0.02 for O_3, 0.92 ± 0.03 for CO, 0.90 ± 0.04 for NO, 0.91 ± 0.03 for NO_x and 0.94 ± 0.03 for O_x.

Table S1: Observed percentage changes (P_{obs}) in air pollutant concentrations (2nd-5th weeks after the lockdown began vs. 2nd-3rd weeks before the lockdown start dates). Changes in mass concentrations of NO_2, O_3, $PM_{2.5}$, NO, PM_{10}, and mixing ratios of O_x and NO_x.

	Beijing	Wuhan	Milan	Rome	Madrid	London	Paris	Berlin	New York	Los Angeles	Delhi
Urban background											
NO_2	-40.5±33.2	-47.3±17.4	-35.1±23.3	-42.2±17.3	-49.0±20.4	-10.1±36.6	-29.8±27.0	-28.1±34.2	-42.4±26.2	-49.5±21.2	-60.2±14.8
O_3	79.6±48.5	166.5±60.5	93.9±65.3	66.1±30.7	16.2±16.1	26.4±20.3	36.1±16.5	57.8±19.8	23.6±24.8	25.9±14.4	86.4±35.8
$PM_{2.5}$	19.2±108.6	-27.7±37.1	N.A.	-30.8±37.9	-38.6±17.2	152.9±165.0	116.0±112.7	N.A.	-30.7±33.0	-33.3±27.8	-19.1±30.3
O_x	2.3±18.3	21.3±25.2	0.6±11.1	6.0±10.7	-1.6±10.3	14.6±10.4	14.0±11.5	27.6±9.9	-3.6±8.1	-5.7±9.3	11.5±12.8
NO	N.A.	N.A.	N.A.	-81.1±12.5	-74.5±8.5	-53.9±38.9	N.A.	-39.2±61.8	N.A.	N.A.	-78.0±13.3
NO_x	N.A.	N.A.	-56.7±18.4	-60.1±14.4	-57.3±15.7	-23.3±33.5	N.A.	-30.0±37.0	N.A.	N.A.	-68.8±12.8
PM_{10}	7.8±81.0	-29.9±32.2	N.A.	-23.8±29.1	-54.1±18.9	116.5±110.	67.0±75.6	35.3±49.8	N.A.	-53.6±36.9	-18.6±30.8
Roadside											
NO_2	-30.8±35.5	N.A.	-38.7±21.9	-41.4±17.4	-46.0±20.1	-53.5±18.9	-34.8±23.3	-29.3±33.1	-37.3±25.9	-50.3±19.7	N.A.
O_3	81.0±67.9	N.A.	155.6±83.2	20.7±11.2	19.5±21.0	134.7±49.8	N.A.	79.4±28.1	N.A.	27.6±19.0	N.A.
$PM_{2.5}$	3.4±94.6	N.A.	N.A.	-17.9±41.8	N.A.	107.6±148.5	45.6±69.9	N.A.	-30.0±49.9	-40.8±28.4	N.A.
O_x	-2.6±18.2	N.A.	0.6±12.3	-5.4±7.3	-4.8±11.2	-6.1±8.6	N.A.	23.5±10.5	N.A.	-11.5±8.2	N.A.
NO	N.A.	N.A.	N.A.	-71.8±15.4	-81.5±12.	-87.6±7.1	N.A.	-53.8±36.0	N.A.	N.A.	N.A.
NO_x	N.A.	N.A.	-52.8±20.8	-57.6±15.6	-65.0±13.4	-74.4±11.0	N.A.	-41.3±33.8	N.A.	N.A.	N.A.
PM_{10}	-5.4±70.1	N.A.	-40.0±21.2	-22.8±25.9	-61.8±9.5	48.4±74.4	25.7±47.4	21.6±48.7	N.A.	-48.5±23.8	-5.4±70.1
Rural											
NO_2	-41.7±28.2	-64.8±18.3	-33.9±19.9	-46.1±22.0	-50.7±9.5	115.8±90.2	99.2±66.7	-25.0±33.9	-40.2±37.6	-47.8±21.7	-43.1±21.0
O_3	76.3±34.8	102.3±43.4	75.5±42.6	29.6±13.8	0.2±13.3	4.2±14.6	16.8±14.2	47.6±14.5	7.0±14.4	5.8±9.9	N.A.
$PM_{2.5}$	9.7±86.3	-14.3±43.	N.A.	-0.1±61.6	-23.7±20.9	164.5±148.7	136.8±144.5	N.A.	-28.0±28.4	-13.3±27.0	-34.2±26.8
O_x	11.9±13.0	23.3±25.9	13.9±13.1	16.2±11.5	-4.2±12.4	11.2±14.9	20.3±13.5	34.9±13.3	0.6±9.2	-6.8±8.8	N.A.
NO	N.A.	N.A.	N.A.	-52.1±33.5	-34.7±3.5	161.4±66.5	N.A.	-74.5±24.0	N.A.	N.A.	-72.4±15.8
NO_x	N.A.	N.A.	-51.7±15.4	-54.1±19.8	-45.9±8.6	124.2±82.4	N.A.	-29.9±32.0	N.A.	N.A.	-52.2±17.5
PM_{10}	6.0±70.2	-26.8±34.2	N.A.	5.6±55.0	-57.3±13.4	161.5±121.9	93.1±90.0	34.0±43.8	N.A.	-34.5±35.2	-22.5±23.9

Note: N.A.: data not available.

Table S2: Deweathered percentage changes (P_{dew}) in air pollutant concentrations (2nd-5th weeks after the lockdown began vs. 2nd-3rd weeks before the lockdown start date). Changes in mass concentrations of NO_2, O_3, $PM_{2.5}$, NO, PM_{10}, and mixing ratios of O_x and NO_x.

	Beijing	Wuhan	Milan	Rome	Madrid	London	Paris	Berlin	New York	Los Angeles	Delhi
Urban background											
NO_2	-33.4±2.2	-43.9±2.2	-27.4±8.3	-33.2±6.1	-49.7±3.1	-18.2±6.0	-33.6±3.3	-25.4±6.0	-23.3±2.0	-23.8±3.4	-52.9±1.4
O_3	28.9±2.0	44.5±3.4	66.8±29.2	55.8±6.7	28.0±3.8	15.8±1.8	22.2±2.4	29.9±3.0	17.4±3.9	14.8±2.2	26.2±5.8
$PM_{2.5}$	-19.3±9.6	-27.0±18.7	N.A.	-16.4±5.2	-43.1±3.4	8.6±8.3	16.5±10.7	N.A.	-21.5±2.6	-18.0±5.4	-12.7±2.8
O_x	-1.1±2.0	1.1±1.4	-1.3±1.0	4.1±1.2	-0.4±1.3	4.2±0.8	2.1±0.6	10.5±0.8	-1.0±1.8	-2.3±1.0	-4.6±3.5
NO	N.A.	N.A.	N.A.	-64.1±4.7	-65.4±3.5	-24.8±6.3	N.A.	-37.1±11.1	N.A.	N.A.	-49.4±1.6
NO_x	N.A.	N.A.	-48.4±6.7	-49.0±5.1	-55.2±3.4	-21.0±5.9	N.A.	-28.2±7.2	N.A.	N.A.	-50.0±1.3
PM_{10}	-8.0±6.5	-24.6±7.7	N.A.	-17.5±2.5	-45.0±4.0	9.0±5.9	3.8±7.4	3.2±1.9	N.A.	-42.7±11.6	-21.1±2.6
Roadside											
NO_2	-21.0±3.0	N.A.	-29.8±9.6	-35.2±7.9	-45.3±3.8	-47.0±6.4	-39.3±4.3	-23.9±10.7	-20.8±6.2	-32.9±4.1	N.A.
O_3	25.4±1.8	N.A.	128.5±41.9	20.3±1.9	28.0±4.9	79.4±5.3	N.A.	46.8±6.2	N.A.	15.0±3.0	N.A.
$PM_{2.5}$	-25.9±5.8	N.A.	N.A.	-9.1±6.8	N.A.	1.0±7.2	0.2±9.1	N.A.	-2.8±1.3	-37.8±4.8	N.A.
O_x	-1.9±2.9	N.A.	-0.0±1.2	-2.2±0.7	-1.2±1.5	-5.7±2.1	N.A.	7.8±0.9	N.A.	-5.7±1.1	N.A.
NO	N.A.	N.A.	N.A.	-62.4±8.4	-66.4±3.8	-71.4±2.1	N.A.	-41.8±16.0	N.A.	N.A.	N.A.
NO_x	N.A.	N.A.	-43.2±8.8	-50.7±8.0	-57.8±3.8	-63.3±2.9	N.A.	-32.7±13.2	N.A.	N.A.	N.A.
PM_{10}	-12.6±3.9	N.A.	-37.5±3.3	-15.1±2.2	-60.3±2.0	-2.3±4.7	-6.4±5.5	-1.8±2.6	N.A.	-41.7±3.7	N.A.
Rural											
NO_2	-35.0±1.8	-49.7±1.2	-32.0±5.9	-39.2±4.2	-52.1±4.8	1.1±2.9	1.0±4.7	-19.7±3.3	-27.9±6.3	-28.9±3.0	-39.2±4.4
O_3	29.4±2.6	27.2±2.4	57.9±6.3	26.6±2.1	6.0±2.8	1.5±0.9	3.3±1.3	19.9±1.7	5.5±3.3	4.5±1.0	N.A.
$PM_{2.5}$	-18.6±7.6	-18.4±18.9	N.A.	7.4±17.6	-28.2±3.0	20.3±11.2	22.6±16.2	N.A.	-10.6±4.1	-19.5±4.8	-20.4±2.5
O_x	4.7±1.6	2.4±1.5	4.2±1.4	14.5±1.0	2.0±2.5	1.2±0.8	4.6±0.8	12.3±1.2	0.6±2.2	-3.0±0.9	N.A.
NO	N.A.	N.A.	N.A.	-47.8±4.8	-35.7±4.9	40.1±8.0	N.A.	-33.8±4.3	N.A.	N.A.	-25.5±3.0
NO_x	N.A.	N.A.	-46.8±4.5	-45.0±6.5	-47.7±5.0	5.7±3.3	N.A.	-23.6±3.1	N.A.	N.A.	-32.9±3.3
PM_{10}	-6.5±4.6	-24.3±14.6	N.A.	2.3±15.7	-57.0±3.4	32.6±9.6	15.8±11.4	8.3±3.6	N.A.	-37.8±6.6	-23.8±3.4

Note: N.A.: data not available.

Table S3: Detrended percentage changes (P^*) in air pollutant concentrations (2nd-5th weeks after the lockdown began vs. 2nd-3rd weeks before the lockdown start date). Changes in mass concentrations of NO_2, O_3, $PM_{2.5}$, and mixing ratios of O_x.

	Beijing	Wuhan	Milan	Rome	Madrid	London	Paris	Berlin	New York	Los Angeles	Delhi
Urban background											
NO_2	-18.5±9.2	-33.9±7.3	-16.3±11.4	-27.1±7.7	-35.2±21.3	-7.7±7.7	-25.8±7.1	-11.3±13.1	-17.0±8.3	-9.9±6.1	-51.0±5.2
O_3	14.8±5.3	21.8±13.6	15.4±37.6	29.8±10.1	11.2±18.3	-1.6±8.1	7.0±5.1	2.6±8.9	5.3±9.8	2.3±5.3	8.2±8.4
$PM_{2.5}$	-2.4±14.7	-15.7±24.8	N.A.	-0.7±9.6	-24.1±18.4	10.9±16.7	27.4±15.3	N.A.	-13.9±6.9	-40.3±26.9	-5.2±4.8
O_x	0.1±7.4	-0.7±4.4	-7.2±5.9	-2.0±2.9	-3.4±3.2	-2.4±1.3	-0.6±2.3	1.8±1.9	-6.4±6.8	-4.1±1.7	-9.8±6.3
Roadside											
NO_2	-9.9±7.6	N.A.	-20.5±13.2	-33.0±9.4	-32.8±19.5	-41.3±12.3	-36.9±6.6	-15.5±17.5	-15.7±13.6	-31.1±6.9	N.A.
O_3	5.8±8.4	N.A.	64.5±55.0	0.3±5.2	-13.2±15.9	40.2±27.7	N.A.	0.1±14.9	N.A.	9.0±5.4	N.A.
$PM_{2.5}$	-2.7±13.7	N.A.	N.A.	2.7±11.1	N.A.	4.5±11.5	7.8±11.4	N.A.	1.5±5.3	-50.8±11.0	N.A.
O_x	1.7±7.5	N.A.	-8.8±5.1	-7.0±2.5	-4.3±2.8	-7.8±5.4	N.A.	-0.9±2.3	N.A.	-6.7±2.2	N.A.
Rural											
NO_2	-26.8±7.0	-28.0±10.7	-15.2±11.7	-35.3±13.1	-36.4±20.7	4.7±8.1	11.5±7.3	-1.6±11.4	-4.8±21.8	-21.9±6.1	-37.3±5.3
O_3	19.5±6.6	10.5±12.3	15.0±15.9	10.4±5.6	0.8±7.3	-5.1±4.6	-2.6±2.7	18.3±21.2	-2.6±8.3	-2.8±4.6	N.A.
$PM_{2.5}$	-3.4±14.9	-10.3±23.6	N.A.	19.9±19.1	-12.2±12.9	19.6±21.3	32.4±22.0	N.A.	-1.0±9.1	-22.7±10.8	-15.3±5.4
O_x	2.4±5.1	-1.6±6.5	-4.7±4.0	1.9±3.8	-3.0±4.8	-2.9±2.6	1.7±1.5	16.3±17.3	-4.7±8.3	-6.4±2.8	N.A.

Note: N.A.: data not available.

Table S4: Site selection and data sources

City	Data source	Weblink	Site type	Site Name (or Site Code)	Quality assurance
Beijing	Official	zx.bjmemc.com.cn/	Urban background	"Dongsi", "Tiantan", "Guanyuan", "Wanshouxigong", "Aotizhongxin", "Nongzhanguan", "Wanliu", "Beibuxincun", "Zhiwuyuan", "Fengtaihuayuan", "Yungang", "Gucheng"	Officially validated
			Roadside	"Qianmen", Yongdingmenbei", "Xizhimenbei", "Nansanhuan", Dongsihuan"	
			Rural	"Fangshan", "Daxing", "Yizhuang", "Tongzhou", "Shunyi", "Chuangping", "Menkougou", "Pinggu", "Huairou", "Miyun", "Yanqing"	
Wuhan	Official	http://106.37.208.233:20035/	Urban background	"1325A","1326A","1327A","1328A","1329A","1330A","1331A","1332A","1333A"	Officially validated
			Rural	"1334A"	
London	Official	https://uk-air.defra.gov.uk/data/data_selector_service?show=auto&submit=Reset&f_limit_was=1	Urban background	"London Bloomsbury", "London Haringey Priory Park South", "London N Kensington", "London Teddington Bushy Park"	Officially validated
			Roadside	"London Marylebone Road"	
			Rural	"Chilbolton Observatory"	
Madrid	Official	http://datos.comunidad.madrid/catalogo/dataset/calidad_aire_datos_historico	Urban background	"28007004"	Officially validated
			Roadside	"28065014", "28074007"	
			Rural	"28016001", "28067001", "28102001", "28133002", "28171001"	
Rome	Official	http://www.arpalazio.net/main/aria/sci/annoincorso/chimici.php	Urban background	"2","8","15","39","48","49","56"	Officially validated
			Roadside	"3", "5", "45", "47", "55", "83", "84", "85"	
			Rural	"14","40"	
Milan	Official	https://www.dati.lombardia.it/browse?q=Dati+sensori+aria&sortBy=relevance&page=1	Urban background	"5517", "5520", "5534", "5547", "5549", "5619", "5630", "5631", "5646", "5710", "5717", "5718", "5825", "5826", "5832", "6300", "6324", "6326", "6346", "6364", "10278", "10279", "10280", "10282"	Officially validated
			Roadside	"5504", "5507", "5531", "5532", "5542", "5551", "5609", "5725", "5812", "5823", "5827", "5834", "5835", "6276", "6320", "6328", "6354", "6356", "6366", "9999", "10001", "10002"	
			Rural	"5548", "5554", "5707", "5721", "5814", "6288", "6338"	
Berlin	Official	https://luftdaten.berlin.de/lqi	Urban background	"042 Neukolln"	Officially validated
			Roadside	"174 Frankfurter Allee"	
			Rural	"032 Grunewald"	
Paris	Official	https://www.airparif.asso.fr/en/telechargement/telechargement-station	Urban background	"ARG", "AUB", "BOB", "CERGY", "CHAMP", "DEF", "EVRY", "GEN", "LOGNES", "MONTG", "NEUIL", "NOGENT", "PA07", "PA12", "PA13", "PA15L", "PA18", "STDEN", "VILLEM", "VITRY"	Officially validated
			Roadside	"A1", "AUT", "BASCH", "BONAP", "BP_EST", "CELES", "ELYS", "HAUS", "OPERA", "RD934", "RN2", "RN6","SOULT"	
			Rural	"RUR-E", "RUR-NE", "RUR-NO", "RUR-SE", "RUR-SO", "RUR_N", "RUR_S"	
New York	"OpenAQ"*		Urban background	"Bayonne", "Newark Firehouse"	Data originated from government- and research-grade sources, and validated manually with official sources
			Roadside	"Fort Lee Near Road"	
			Rural	"Chester", "Flemington", "Rutgers University"	
Los Angeles	"OpenAQ"	https://openaq.org/#/?_k=etobqa	Urban background	"Compton", "Lancaster-Division"	
			Roadside	Los Angeles - N. Mai	
			Rural	"Glendora - Laurel", "LAX-Hastings"	
Delhi	Official	https://app.cpcbccr.com/ccr/#/caaqm-dashboard-all/caaqm-landing	Urban background	"Nehru Nagar"	Officially validated
			Rural	"Sonia Vihar", "Sri Aurobindo Marg"	

*OpenAQ data are from official sources although they are not necessarily validated before being released. We evaluated the officially released air quality data from Los Angeles (after the data analyses in this study) with OpenAQ data; they are highly correlated (both slope and correlation coefficient are either equal to or close to 1). This demonstrates that the sources of data are reliable.

Auxiliary Data Table S1. Original air quality and meteorological data, as well as deweathered air quality concentrations. Available from https://github.com/songnku/COVID-19-AQ
